# The Effects of External Lower Limb Weight or Pressure Application on Human Knee Joint Proprioception in Resting and Fatigue Conditions: A Randomized Trial

**DOI:** 10.3390/jfmk11030262

**Published:** 2026-06-30

**Authors:** Elmina-Eleftheria Roditi, Themistoklis Tsatalas, Giorgos K. Sakkas, Ioanna Giannopoulou, Yiannis Koutedakis, Giannis Giakas, Christina Karatzaferi

**Affiliations:** 1 Experimental Physiology & Myology Unit—Center for Research and Evaluation of Human Performance, Department PE & Sports Science, University of Thessaly, Karyes, 42100 Trikala, Greece; elminion@yahoo.com (E.-E.R.); gianioanna@hotmail.com (I.G.); y.koutedakis@uth.gr (Y.K.); 2 Biomechanics and Ergonomics Unit—Center for Research and Evaluation of Human Performance, Department PE & Sports Science, University of Thessaly, Karyes, 42100 Trikala, Greece; ttsatalas@uth.gr (T.T.); ggiakas@uth.gr (G.G.); 3 Lifestyle Medicine Unit—Center for Research and Evaluation of Human Performance, Department PE & Sports Science, University of Thessaly, Karyes, 42100 Trikala, Greece; gsakkas@uth.gr; 4 School of Life and Health Sciences, Nicosia University, Nicosia CY-2417, Cyprus; 5 Faculty of Education, Health and Wellbeing, University of Wolverhampton, Wolverhampton WV1 1LY, UK

**Keywords:** proprioception testing, knee joint position sense, external weight, pressure application, neuromuscular fatigue, women, men, open kinematic chain

## Abstract

**Objectives**: Knee proprioception is affected by many biomechanical and physiological factors. Often, during training, rehabilitation, or specific sport requirements, weight or pressure is applied to the foot. However, it is not clear if such applications affect knee proprioceptive acuity. This study examined whether the application of an external weight (3 kg) or of pressure (120 mmHg) at a level above the ankle joint would affect knee proprioception in an open kinematic chain movement in resting or fatigue conditions. **Methods:** Participants included active young men (*n* = 7) and women (*n* = 15), aged 21–34 years, without prior knee injury. Women were tested in their follicular phase. An isokinetic dynamometer was used to evaluate knee joint repositioning. Three knee angles were targeted (30°, 45°, 60°) before and after localized muscle fatigue. A three-way ANOVA analysis with repeated measures and one independent variable (gender) was performed. **Results:** Analysis showed that ankle weight application positively influenced knee joint proprioceptive acuity resulting in an overall reduction in knee joint angular error for both genders, with the “corrective” effect most evident at 45° and 60° knee joint angles (*p* < 0.05), whether in the rested or fatigued state. The application of pressure however improved knee proprioceptive performance in men (*p* < 0.05), in both the rest and fatigue states, but not in women (ns tendency). **Conclusions:** Application of a small weight at the ankle level significantly improved proprioceptive knee joint acuity. These findings may have wider applicability by allowing the development of specific preventive measures towards safeguarding weaker and/or less resilient players.

## 1. Introduction

Proprioception is a fundamental component of neuromuscular performance and postural control [1]. It is defined as an individual’s awareness of the position and movement of body parts in space, the detection of rapid changes in the mechanical state of tissues, and even the sense of balance [1,2]. This complex system encompasses both spinal and cortical projections and can be divided into two primary components: the sense of joint position and the sense of limb movement, or kinesthesia [2]. Proprioceptive information is not generated by individual receptors but by a combination of afferent inputs from the skin, muscles, and joints [1,3]. Consequently, it plays a vital role in avoiding abnormal movements, such as extreme extension or flexion, thereby preventing injuries [4].

Proprioception is commonly quantified using joint position sense threshold tests, where subjects detect passive movement or replicate target angles [5,6,7]. The knee is frequently assessed as it is a primary joint in human movement [8,9]. Active angle reproduction tests, which measure the ability to actively return a limb to a target angle, are particularly relevant for field applications. Results are typically reported as Absolute Angle Error (AAE) and, to a lesser extent, as Signed Angle Error (SAE) [8].

Acuity is affected by factors such as age, gender, and fatigue [10,11]. For instance, knee position sense is more accurate in a sitting versus a prone position [12] and in closed versus open kinematic chain (OKC) conditions [10]. In OKC movements, muscle fatigue has been shown to impair proprioception in both men [13] and women [11]. Furthermore, OKC movements generate higher tension in the ACL during extension and the PCL during flexion compared to closed-chain movements [14,15]. Given that women experience ACL injuries two to eight times more frequently than men [16,17], understanding these dynamics is crucial. Previous work by our group [11] found that fatigue impairs accuracy in young women during OKC tasks, but it is unknown if these findings apply to men.

External factors also play a significant role. Athletes often use ankle taping for stability [18,19,20], while ankle weights are used for strengthening in sports and dance [21]. Additionally, equipment like steel-toe boots can apply substantial weight to the lower limb. Taping may act as a proprioceptive aid by enhancing cutaneous input to the CNS [22]. However, it remains unclear whether ankle weights or taping influence knee proprioception and how fatigue modulates these effects in both genders.

The present study aimed to examine whether the application of external weight or pressure at the ankle level could affect knee joint repositioning accuracy, in comparison to no application; whether fatigue would compromise proprioception; and whether any effects would be angle-dependent in young physically active women and men.

## 2. Materials and Methods

### 2.1. Subjects

The sample was 22 athletes (15 women, 7 men) aged 21–34 years, active in team sports or contemporary dance, located in the city of Trikala, Greece. Exclusion criteria comprised less than 3 years of training, chronic disease, lower extremity injury/pain within the past year, and for women, gynecological issues or irregular cycles [11]. The study was approved by the University of Thessaly DPESS Ethics Committee (code 3-1/12122012-B), and all participants provided written informed consent. Patients and the public were not involved in the design, conduct, or reporting of this trial. All experimentation took place at the core laboratory (CREHP) of the Department of P.E. and Sport Science, School of P.E., Sport Science and Dietetics, University of Thessaly.

### 2.2. Study Design

**Figure 1:**
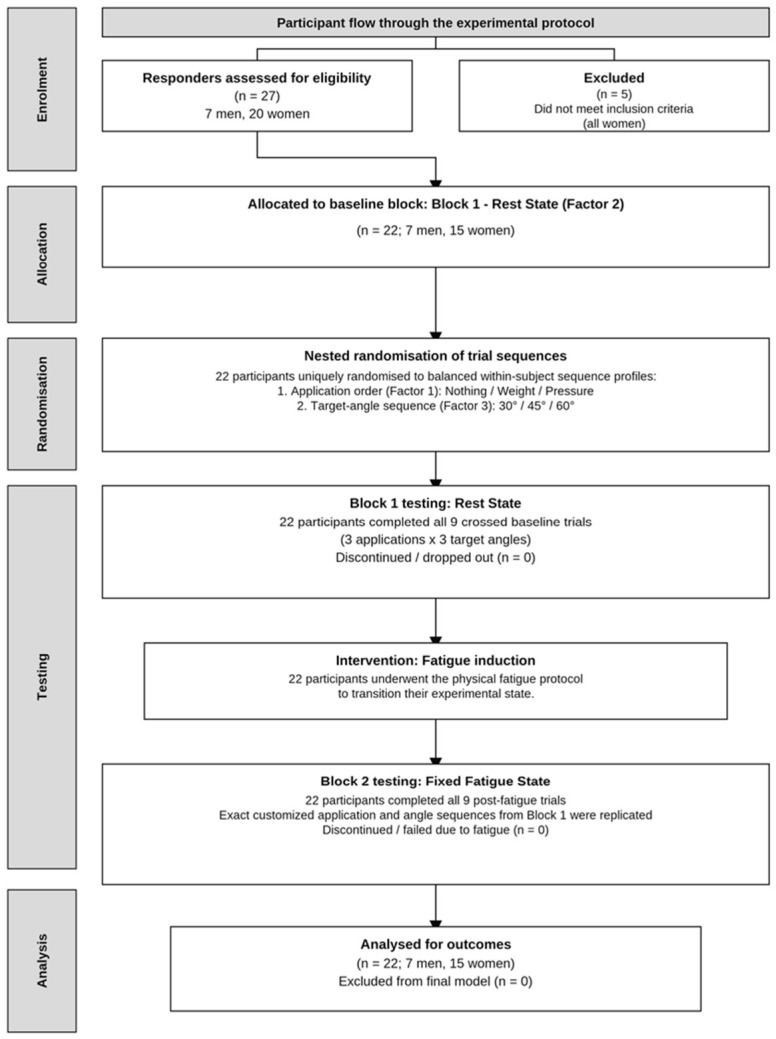
Participant flow diagram illustrating enrolment, nested sequence randomization, block testing phases, and complete final data analysis (constructed taking into account the CONSORT 2025 Statement [24] and the PRISMA 2020 style [25]. Refer to the Supplementary File for more study design details.

This was a randomized, counterbalanced, repeated measures design. Of 27 initial responders (7 men, 20 women), 22 (7 men, 15 women) met the criteria (Figure 1). One week prior, baseline height and weight were recorded [23] and dominant limbs identified. Experimental sessions occurred between 08:00 and 12:00 over three visits (48 h apart). Women were tested during their follicular phase (days 7–14). Participants performed knee joint position sense evaluations at 30°, 45°, and 60° target angles under three randomized conditions—“nothing”, “weight” and “pressure” applications—both before and after a fatigue protocol. Both condition and angle order were randomized to prevent order effects.

### 2.3. Instrumentation

Participants warmed up (for 7 min, at 80 W) on a Monark cycle ergometer (834E, MONARK, Vansbro, Sweden). A CYBEX NORM^®^ isokinetic dynamometer (Cybex Norm, Lumex Corporation, Ronkohoma, NY, USA) was utilized to determine the dominant limb, assess knee position sense via standardized protocols [11,26], and execute the fatigue protocol [11,27].

### 2.4. Preliminary Assessments

In preparation for the study, we evaluated the duration and regularity of the menstrual cycle of women participants for two months prior to study participation. This monitoring phase allowed us to plan their testing sessions [11]. During a familiarization visit, one week before the start of experimental data collection, subjects’ basic characteristics (body weight in kg and height in m) were noted using scales and a stadiometer [SECA, Vogel & Halke, Hamburg, Germany]; body mass index (BMI) was calculated in kg/m^2^. The weight was recorded in each subsequent visit.

#### Determination of Dominant Lower Limb

Dominant limb determination occurred one week pre-experiment using a CYBEX NORM^®^ dynamometer to evaluate maximum torque. Following a 7 min warm-up at a speed of approx. 70 rpm and 5 min guided stretching, subjects performed five maximal concentric knee extension/flexion repetitions at 120°/s [27] for each limb; the limb that produced higher peak torque was defined as dominant. Anatomical zero was set at full extension, with a total range of motion of 110° (5° to 115°).

### 2.5. Experimental Sessions

Subjects returned 2–3 days post-preliminary assessment for the first experimental session to evaluate knee position sense [9] under rested and fatigued states. Three randomized ankle conditions were applied to the dominant limb: “nothing” (no load), “weight” (3 kg), or “pressure” (120 mmHg). Specifically, under “weight” application, a 3 kg ankle weight (AMILA^®^ neoprene band, ELDICO SPORT S.A., Athens, Greece) was secured circumferentially around the ankle joint, centered over the lateral malleolus; under “pressure” application, an inflatable cuff was secured circumferentially around the ankle joint, centered over the lateral malleolus, inflated and maintained to 120 mmHg during the time needed to assess proprioception for the three knee target angles. Randomization was done by a member of the team using a concealed block randomization sequence. No ankle weights or pressure cuffs were worn during the fatigue induction protocol. Subjects wore the same personal athletic footwear and were not aware of the order.

#### 2.5.1. Position Sense Assessment

The experimental protocol assessed knee joint position sense at three target angles (30°, 45°, and 60°), with full extension defined as 0°. To eliminate order effects, the presentation of these angles was randomized both within and between participants. The same two investigators delivered the protocol. Subjects were positioned in a seated posture with a 120° hip angle. The procedure involved the researcher passively moving the participant’s lower limb from a 90° flexion starting position to the randomized target angle. This position was held for 10 s to allow for proprioceptive encoding before returning the limb to the 90° start position.

Participants were then instructed to actively replicate the target angle. Once subjects were satisfied with their perceived position, they maintained it for 2 s. Three trials were recorded per angle, and the average was used for analysis. Data were processed to determine the primary outcome, Signed Angle Error, representing the deviation from the target (e.g., 29° for a 30° target resulted in a −1° error, i.e., stopping short of the target angle, undershooting; 31° for a 30° target resulted in a +1° error, i.e., overshooting). Additionally, performance was calculated as a percentage of the target angle achieved (e.g., 33° for a 30° target equated to 110%). This normalization allowed for a comparative evaluation of accuracy across different target positions.

#### 2.5.2. Muscle Fatigue Protocol

For the fatigue protocol the participants performed maximal repetitions until the recorded torque, over three consecutive repetitions, decreased to 50% of their maximum torque. The protocol included continuous maximum repetitions of extension and flexion of the knee joint at the isokinetic dynamometer with concentric manner (angular velocity 120°/s) [27]. We questioned participants for possible pain or soreness intensity using the standardized Delayed Onset Muscle Soreness (DOMS) scale (from 0 = “no pain” to 10 = “worst possible pain”).

### 2.6. Statistical Analysis

Power analysis was performed using the open-source software G*Power (3.1.9.2) to calculate the minimum number of participants required to achieve reasonable power (>80%). A post hoc analysis revealed that in one of the main parameters related to the aims of the study (angle error at 60° target angle), we had enough power to detect statistically significant differences between the pre- and post-fatigue intervention comparisons (*n* = 22, a = 0.05, power = 0.99). The baseline data analyst was blinded to the design (and is not a member of the authors list). Data normality was confirmed via the Shapiro–Wilk test, with results presented as Mean ± SD. A 3-way repeated measures ANOVA assessed three factors (to examine main effects and interactions of) in the total sample: 1. application (nothing, weight, pressure), 2. state (pre- vs. post-fatigue), and 3. target angle (30°, 45°, 60°). Gender served as the between-subjects independent variable. Relationships between performance and variables were analyzed using Pearson correlation coefficients. Statistical significance was set at *p* ≤ 0.05, utilizing Bonferroni post hoc adjustments. Effect sizes were reported as Partial Eta Squared (ηp^2^), where 0.01, 0.06, and >0.14 represented small, medium, and large effects, respectively. We used the statistical software SPSS v. 29.0 (SPSS Inc., Chicago, IL, USA).

## 3. Results

All 22 participants (15 women, 7 men) completed the study without discomfort, pain or missing data. No changes were made to the trial design, eligibility criteria, outcomes, or main analyses after the trial commenced. Initial analysis confirmed a normal distribution for the majority of variables (Shapiro–Wilk, *p* > 0.05) justifying the use of parametric statistics. Participants’ physical characteristics are summarized in Table 1; body weight remained stable throughout the sessions.

Proprioceptive performance was evaluated using signed angular error and percentage deviation following three different applications, before and after fatigue, across three target angles (30°, 45°, 60°). Analysis of the signed error revealed a significant main effect for Application (F(2, 19) = 12.40, *p* < 0.001, ηp^2^ = 0.566). Post hoc comparisons showed that while both the control and pressure (taping) conditions were associated with larger angular errors, the weight application significantly improved accuracy (*p* < 0.01).

A significant main effect was observed for State (F(1, 20) = 13.66, *p* = 0.001, ηp^2^ = 0.406), with muscle fatigue consistently increasing angular error across all conditions and target angles (Table 2). Finally, a main effect for Target Angle was identified (F(2, 19) = 67.54, *p* < 0.001, ηp^2^ = 0.877); specifically, more flexed knee positions resulted in significantly larger errors (*p* < 0.01), independent of gender, state, or application condition.

Analysis revealed no significant interaction between Application and State (*p* = 0.394) or Application and Target Angle (*p* = 0.943). However, the State × Target Angle interaction was significant (F(2, 19) = 11.49, *p* < 0.001, ηp^2^ = 0.547), with fatigue specifically worsening performance at the 45° (*p* < 0.05) and 60° target angles (*p* < 0.01). Between-subjects analysis identified a significant Gender × Application interaction (F(2, 19) = 9.77, *p* = 0.001, ηp^2^ = 0.507); under the “weight” condition, women exhibited larger average errors (−2.6°) in the opposite direction to men (+0.8°). Additionally, the Gender × Target Angle interaction was significant (*p* < 0.01). While both sexes performed similarly at 45° and 60°, men showed larger deviations at 30° (+2.9°) compared to the minimal error observed in women (+0.05°).

When data were expressed as a percentage of the target angle, the main effects for Application, State, and Angle remained significant (*p* < 0.01). Notably, the State × Target Angle interaction observed in the signed error analysis was no longer significant in percentage terms (*p* = 0.150).

A significant triple interaction was found between Gender × State × Target Angle (F(2, 19) = 5.20, *p* < 0.05, ηp^2^ = 0.354). Post hoc analysis showed that fatigue significantly impaired accuracy in women across all target angles (*p* < 0.05). In contrast, men’s accuracy was significantly affected by fatigue only at the 60° target angle (rest: −8.1% vs. fatigue: −13.8%).

**Figure 2:**
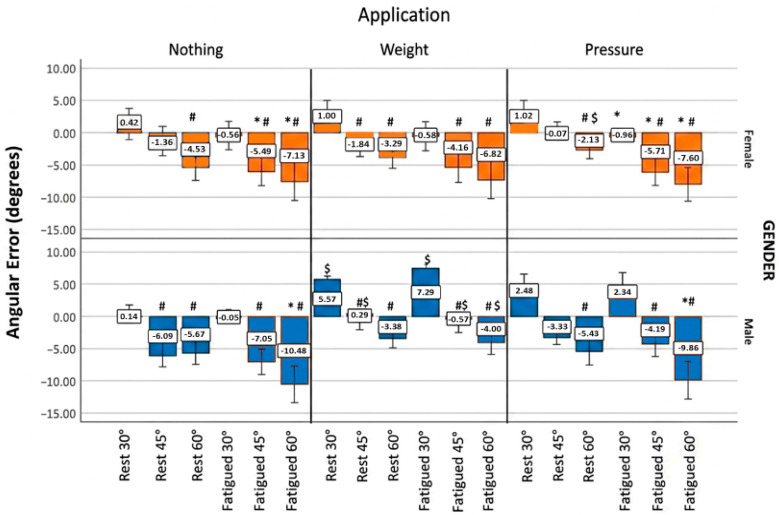
Deviation from the intended “target angle” (30°, 45°, 60°), in the rest and fatigued states, with the three different applications (nothing-applied, weight, pressure), examined in women (*n* = 15) and men (*n* = 7). Values in signed degrees. * Significant differences *p* < 0.05 from rest, at the respective target angle. **#** Significant differences *p* < 0.05, compared to 30° target angle performance, at the respective state. 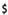 Significant differences from “nothing-applied”, at the respective target angle and state.

Overall, men demonstrated numerically greater deviations from the target angles than women across all applications (Figure 2), with the direction of error frequently reversing in the male cohort.

**Figure 3:**
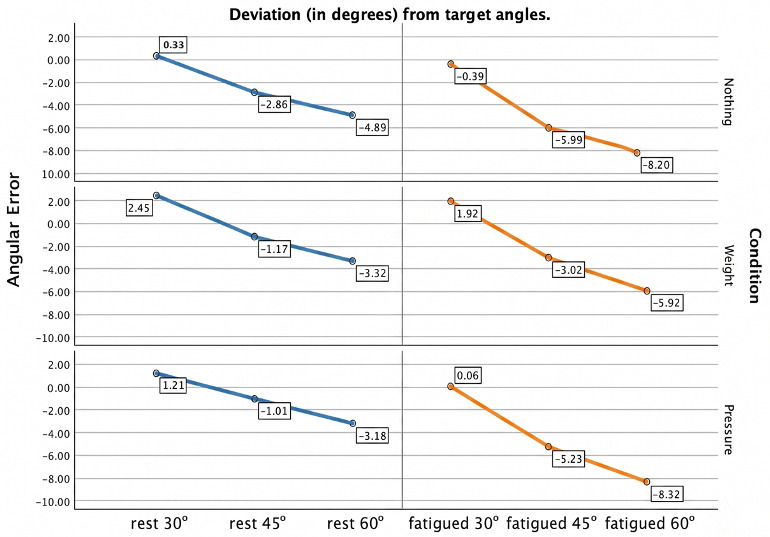
Deviations from the target angle (*N* = 22) in all conditions at rest (blue) or fatigue (orange).

If we view the results without gender separation, we notice a similar ‘modulation’ of the replication performance at the various angles for the three conditions (Figure 3), with a larger magnitude of signed angular error (i.e., deviation from target) when targeting 60°, as earlier commented. Again, the largest angular error for all three angles was observed after fatigue (Figure 3).

Correlation analysis of angular error (signed degrees and percentages) revealed significant (*p* < 0.05) weak-to-strong correlations (r = 0.38 to 0.88) across the three application conditions (nothing, pressure, weight) and between rest and fatigue states at most angles. Notably, average error pre- and post-fatigue was highly correlated (r = 0.91) for the entire sample and both genders (*p* < 0.05).

However, specific exceptions occurred: no correlation existed between “nothing” and “weight” at 60° during fatigue (Condition Mismatches); no correlation was found between resting and fatigued states at 45° (pressure) or 60° (weight) (State Mismatches).

Regarding physical attributes, body weight did not correlate with target error. Peak torque showed no significant relationship with error in men. Conversely, in women, peak torque correlated strongly and positively with average error at rest (r = 0.59, *p* < 0.05). Additionally, some significant correlations (mostly negative) appeared in women between peak torque and errors. Specifically, at 45° there was a correlation of peak toque in women with error post-fatigue and under the “nothing” application (r = 0.55, *p* < 0.05), and under resting and pressure application (r = 0.69, *p* < 0.05); at 60° there was a correlation of peak torque in women with error under the “nothing” and “pressure” applications at rest (r = 0.55 and r = 0.54 respectively, *p* < 0.05) and with fatigue under “pressure” application (r = 0.56, *p* < 0.05).

## 4. Discussion

This study examined the effects of biomechanical and physiological factors on knee proprioception during an open kinematic chain (OKC) movement in both women and men. The primary goal was to understand how external stimuli, such as weight and pressure, interact with internal states like muscle fatigue to influence joint position sense (JPS). Our findings demonstrate a complex “modulation” of replication performance, where external aids generally enhance accuracy while fatigue impairs it, particularly for more acute target angles (notably 60°). Perhaps targeting the 30° knee angles predisposed for different performance of the proprioceptive control compared to targeting 45° and 60° knee target angles. Muscle spindles and Golgi tendon organs provide information about muscle length/stretch and tension levels, which helps to determine the direction of movement and the position of the body, as well as reflex actions [28].

The application of a 3 kg weight to the ankle joint appeared to have a positive effect on knee joint proprioception. We observed an overall reduction in angular error for both genders, with a “corrective” effect most evident at 45° and 60° knee joint angles, lowering the error range by 1% to 6%. Interestingly, in men at rest, the application of weight did more than just reduce error magnitude in comparison to “nothing applied”; it shifted the error’s direction. Without weight, men averaged an error of −7.5% (target underestimation), which transitioned to a +4.5% average error (overestimation) with the weight applied. A similar shift occurred during fatigue (−11% to +4.7%). This suggests that the external load provided a potent sensory anchor. This finding is highly consistent with Kim et al. [21], who demonstrated that ankle weights improved JPS in both young and older populations. The mechanism likely involves the augmentation of afferent cues; the added mass increases tension on muscle spindles and Golgi tendon organs, providing the central nervous system (CNS) with more robust data to rectify performance. As Stillman and McMeeken [29] reported, increased joint loading—even via small weights—can activate neurophysiological mechanisms that enhance positional awareness. This implies that small ankle weights, often used for strengthening in athletic or dance contexts [30], can be used safely without degrading proprioceptive acuity; in fact, they may improve it for those with poor baseline ability.

Regarding the application of 120 mmHg pressure above the ankle joint, we observed statistically significant beneficial effects on knee joint repositioning in men for both rest and fatigue states, with benefits reaching approximately 5% in fatigue. Interestingly, this pressure application did not significantly influence proprioceptive ability in women; however, those in the present study were the participants with overall less baseline error. The use of circumferential pressure likely stimulated cutaneous mechanoreceptors, which appeared to serve as a secondary source of positional information. Previous research by You et al. [31] found that similar ankle pressure improved postural stability in healthy subjects with low proprioceptive acuity. Furthermore, Stoffel et al. [19] noted that ankle taping provided protective benefits to the knee by reducing internal rotation moments. Guidelines often emphasize that pressure should not hinder movement [32], and the 120 mmHg (1.6 N/cm^2^) level used here, comparable to ski boot pressures studied by Hermann et al. [33], appeared to be an effective threshold for promoting knee JPS without being restrictive.

The induction of muscle fatigue led to a universal increase in angular error for both women and men. This deficit was most pronounced at the more acute (flexed) angles, specifically at 45° and 60°. Fatigue is one factor that can decrease the proprioceptive acuity [34], and therefore contributes as a risk factor to joint injury [35,36]. Our results align with our previous study [11], showing the lowest proprioceptive acuity at 60° during fatigue in women, and with findings in active men where absolute, but not relative, error increased at 30° under similar fatigue protocols [37]. One should also consider that the knee’s intra-articular structures (ACL, PCL, medial and lateral menisci, and the synovial membrane) contain mechanoreceptors whose population density varies; it has been found to be particularly high in areas associated with reaching a joint’s extreme positions, and less dense in middle-of-the-range positions [38]. Overall, sensory information is expected to vary depending on the knee joint angle, which could explain, together with the available mechanoreception, the angle dependency of proprioceptive acuity observed by us in this study and previously [11]. Furthermore, others [8] observed decreased acuity in both sexes following lower limb fatigue. Notably, the literature has not yet identified specific functional differences in proprioceptive organs, such as Golgi tendon organs, between open and closed kinematic chains [39]; thus, we can assume transference to CKC movements.

The consistent degradation of accuracy when targeting 60° under fatigue highlights a major safety risk for those requiring operational resilience, as fatigue likely “muffles” the sensory feedback loop. Given that strength may influence acuity [40,41], we analyzed correlations between peak torque and error across conditions. In men, we observed a non-statistically significant tendency for the stronger participants to produce less error. In women, weaker participants produced larger errors with a negative sign while stronger women produced smaller-in-magnitude errors, often with a positive sign, independently of the fatigue state. We have not observed similar findings elsewhere in the literature (which mostly reports absolute errors), thus we cannot explain, with the current data, why the two gender groups presented with a differing extent and direction (sign) of error (with women undershooting more, i.e., negatively signed error, the weaker they were). Perhaps there is an interaction between neurophysiological and biomechanical parameters that necessitates further exploration.

While some reports on non-athletic older adults show women performing worse in CKC tests [42], those differences are often linked to muscle weakness rather than sex traits. In our young, active sample, men demonstrated significantly worse accuracy than women across all target angles and conditions. This represents one of the first mixed-gender observations in the open kinematic chain (OKC) literature, furthering the need to compare neuromuscular strategies between sexes. We hypothesize that some differences may relate to training history and training age [43,44,45]. In our case, female participants had histories of sports requiring high precision (e.g., gymnastics, dance) and may have achieved a higher peak proprioceptive acuity than the men of our sample, despite similar levels of recent general physical activity.

Our correlation analysis further supported the consistency of these patterns. We found moderate-to-strong correlations between resting and fatigued states in most conditions. However, the lack of correlation at 45° (under the pressure application) and 60° (under the weight application) between these states suggests that high-flexion angles combined with external stimuli create a unique sensory environment that fatigue disrupts more sporadically. Interestingly, peak torque correlated positively and strongly with average error in women at rest, but not in men. This suggests that in the female sub-cohort, muscle strength may have played a more direct role in proprioception during non-fatigued states. No correlation was observed between body weight and error, suggesting that the JPS deficits were rather neuromuscular-driven.

Practical implications: A first practical implication of this research may be that small ankle weights or moderate levels of pressure might serve as a viable proprioceptive aid for otherwise healthy populations with suboptimal joint sense, “improving” the sensory-motor loop through supplemental afferent data. Secondly, the vulnerability identified at 60° under fatigue may necessitate targeted “fatigue-proofing” neuromuscular training to mitigate risks like ACL tears. Current international soccer regulations allowing five substitutions are vital for injury prevention. However, as 10% fewer substitutions occur in women’s matches compared to men’s [46], federations should actively promote more substitution opportunities for women to reduce fatigue-induced knee injuries.

Study limitations: This study acknowledges certain limitations, as we did not assess knee proprioception during closed kinematic chain or passive movements, nor did we record electromyographic activity. However, the chosen active open kinematic chain (OKC) movement is highly relevant to athletic performance, dance, and daily life. OKC exercises are also critical in rehabilitation, often being superior or equal to closed-chain movements for managing knee laxity [15].

While variable training histories (sports vs. dance) may have influenced results, we maintained strict inclusion criteria: at least three years of training and no prior knee injuries. Additionally, we rigorously monitored menstrual cycles for two months to ensure stability—a step often overlooked [47]—testing women specifically in the follicular phase. The small number of male participants (*n* = 7) limits the robustness of any gender-related conclusions; our study was not designed for gender comparisons, and our finding of a Gender × Application interaction effect should be considered with caution, as it requires replication in a larger and balanced sample. It should however be noted that, despite interindividual differences in proprioceptive acuity, overall subjects followed a similar pattern of response across the conditions.

We did not examine a fatigue–proprioception dose–effect relationship but used the same knee extensors MVC % decline threshold as a criterion for inducing localized muscle fatigue in all participants. Still, we minimized the likelihood of injury by employing concentric contractions. However, the perception and persistence of fatigue can be variable and it would depend on interindividual differences regarding fitness levels and types of training or overall physical activity levels; thus, our observations may not have relevance for whole-body fatigue or other muscle-fatiguing situations. We focused on knee extension proprioceptive performance, but evidence suggests absolute error may be greater in knee flexion [12], and that a fatigue protocol like ours can significantly affect both extensors and flexors [34]. Thus, our results might extend to flexion deficits. Future research should concurrently assess knee and ankle proprioception to enhance safeguarding protocols for injury-prone populations.

## 5. Conclusions

In conclusion, our results showed that external lower limb weight or pressure application at the level of the ankle can have a measurable influence on knee joint proprioception, towards overall improving proprioceptive acuity, in both resting and fatigue states; that fatigue lowers proprioception; and that the larger knee joint repositioning errors were noted at the more acute (flexed) knee joint target angles.

Muscle fatigue significantly reduced active knee repositioning accuracy in both genders, particularly at flexed angles (45° and 60°). The application of a 3 kg ankle weight or circumferential pressure effectively mitigated these deficits, enhancing proprioceptive acuity primarily in men during the fatigued state. Notably, gender differences in the error direction (women more negative) or magnitude (larger in men) highlight the need for sex-specific investigations.

These findings also suggest hypotheses for future investigation in older or clinical populations, but direct applicability cannot be assumed without further studying small ankle weights or pressure aids, which may rectify positional sense for such populations. Coaches and trainers should implement targeted preventive measures for athletes performing rapid transitions from acute to obtuse knee angles, especially those lacking resilience. Furthermore, these results can optimize human resource deployment in physically demanding occupational settings to ensure safety.

## Figures and Tables

**Table 1:** Characteristics of the study participants (*N* = 22). Data values are presented as Mean and Standard Deviation.

	Women *n* = 15	Men *n* = 7	All Participants *N* = 22
Age (years)	25.13 ± 4.29	25.00 ± 4.72	25.09 ± 4.31
Weight (kg)	60.25 ± 9.63	75.34 ± 7.94	65.05 ± 11.47
Height (m)	1.64 ± 0.58	1.74 ± 0.72	1.67 ± 0.79
BMI (kg/m^2^)	22.35 ± 3.08	24.65 ± 1.77	23.08 ± 2.90

BMI = body mass index, capital *N* stands for the total sample size of 22 participants, while small *n* stands for the subgroup of women or of men.

**Table 2:** Deviation (in degrees) from intended target angles, before and after the fatigue protocol with the three different examined applications (*N* = 22).

Target Angles	Application	State	
		REST	FATIGUE
30°	Nothing-applied	0.33 ± 6.24	−0.39 ± 6.36 *
	Weight	2.45 ± 4.95 §	1.92 ± 6.60 *§
	Pressure	1.21 ± 5.38 §	−0.06 ± 6.45 *§
45°	Nothing-applied	−2.86 ± 6.01 **#**	−5.99 ± 5.72 *#
	Weight	−1.17 ± 4.65 **#**§	−3.02 ± 6.42 *#§
	Pressure	−1.01 ± 6.81 **#**§	−5.23 ± 4.76 *#§
60°	Nothing-applied	−4.89 ± 4.62 **#**	−8.20 ± 5.03 *#
	Weight	−3.32 ± 4.60 **#**	−5.92 ± 5.56 *#
	Pressure	−3.18 ± 4.39 **#**§	−8.32 ± 5.49 *#§

Data are expressed as Mean ± SD (*N* = 22). * Significant differences *p* < 0.05 from rest, **#** significant differences compared to 30° target angle. § “from nothing-applied”.

## Data Availability

The original contributions presented in the study are included in the article and the Supplementary Files; further inquiries can be directed to the corresponding author.

## Supplementary Materials

The following supporting information can be downloaded at: https://www.mdpi.com/article/10.3390/jfmk11030262/s1.
