# Supplementary material for: The Effects of External Lower Limb Weight or Pressure Application on Human Knee Joint Proprioception in Resting and Fatigue Conditions: A Randomized Trial
Source: J Funct Morphol Kinesiol. 2026 Jun 30;11(3):262. doi: 10.3390/jfmk11030262 (PMC13398068; doi:10.3390/jfmk11030262)
Supplement: Supplementary file 1 [file jfmk-11-00262-s001.zip › jfmk-4345704-supplementary.pdf]

Supplementary File

-Statistical Results

Note: Effect sizes are reported as Partial Eta Squared (ηp2), where 0.01, 0.06, and >0.14 represented small, medium, and large effects, respectively.

<u>Main effects</u>

-Application (three levels: 1. Nothing 2. Weight 3. Pressure)

Analysis of the signed error revealed a significant main effect for Application ($F(2,19)=12.40, p<0.001, \eta p2=0.566$).

Estimates			95% Confidence Intervals	
	Mean	Standard Error	Lower Bound	Upper Bound
1.	-3.986	1.145	-6.375	-1.598
2.	-0.875	0.817	-2.579	0.829
3.	-2.784	0.996	-4.862	-0.706

Post-hoc comparisons showed that the weight application significantly improved accuracy (p<0.05)

- State (two levels: 1. Rest, 2. Fatigue)

A significant main effect was observed for State ($F(1,20)=13.66, p=0.001, \eta p2=0.406$), with muscle fatigue consistently increasing angular error across all conditions and target angles.

Estimates			95% Confidence Intervals	
	Mean	Standard Error	Lower Bound	Upper Bound
1.	-1.448	0.867	-3.257	0.360
2.	-3.648	1.077	-5.895	-1.401

Muscle fatigue significantly increased the angular error across all conditions and target angles.

- Target Angle (three levels: 1.Thirty degrees, 30°, 2.Forty=-five degrees, 45°, 3. Sixty degrees, 60°)

A main effect for Target Angle was identified ($F(2,19)=67.54, p<0.001, \eta p2=0.877$)

Estimates			95% Confidence Intervals	
	Mean	Standard Error	Lower Bound	Upper Bound
1.	1.502	1.104	-0.802	3.806
2.	-3.287	1.017	-5.408	-1.166
3.	-5.860	0.891	-7.718	-4.002

More flexed knee positions resulted in significantly larger errors ($p<0.01$), independent of gender, state, or application condition.

<u>Interaction Effects</u>

-Application X State

Analysis revealed no significant interaction between Application and State ($p=0.394$)

-Application X Target Angle

Analysis revealed no significant interaction between Application and Target Angle ($p=0.943$).

-State (2 levels) X Target Angle (3 levels)

The State × Target Angle interaction was significant ($F(2,19)=11.49, p<0.001, \eta p2=0.547$),

Estimates				95% Confidence Intervals	
State	Angle	Mean	Standard Error	Lower Bound	Upper Bound
1.	1.	1.773	1.043	-0.402	3.948
	2.	-2.046	1.078	-4.295	0.203
	3.	-4.072	0.839	-5.822	-2.322
2.	1.	1.231	1.249	-1.375	3.837
	2.	-4.528	1.145	-6.917	-2.139
	3.	-7.648	1.019	-9.775	-5.522

Between-subjects analysis identified a significant Gender × Application interaction ($F(2,19)=9.77, p=0.001, \eta p2=0.507$); under the "weight" condition, women exhibited larger average errors (-2.6∘) in the opposite direction of men (+0.8∘).

Estimates				95% Confidence Intervals	
Gender	Application	Mean	Standard Error	Lower Bound	Upper Bound
1. (women)	1.Nothing	-3.107	1.292	-5.802	-0.413
	2. Weight	-2.615	0.921	-4.537	-0.693
	3.Pressure	-2.552	1.124	-4.896	-0.207
2. (men)	1.Nothing	-4.865	1.891	-8.809	-0.921
	2. Weight	0.865	1.349	-1.949	3.678
	3.Pressure	-3.016	1.645	-6.448	0.416

Additionally, the Gender × Target Angle interaction was significant ($p=0.005$).

Estimates					95% Confidence Intervals	
Gender	Angle	Angle	Mean	Standard Error	Lower Bound	Upper Bound
1.Women	1.	2.	3.141	0.508	1.815	4.468
		3.	5.311	0.884	3.002	7.620
	2.	1.	-3.141	0.508	-4.468	-1.815
		3.	2.170	0.811	0.052	4.288
	3.	1.	-5.311	0.884	-7.620	-3.002
		2.	-2.170	0.811	-4.288	-0.052
2.Men	1.	2.	6.436	0.743	4.494	8.378
		3.	9.413	1.294	6.033	12.793
	2.	1.	-6.436	0.743	-8.378	-4.494
		3.	2.977	1.187	-0.123	6.077

	3.	1.	-9.413	1.294	-12.793	-6.033
		2.	-2.977	1.187	-6.077	0.123

- CONSORT 2025 Checklist*

Section / Topic	No	CONSORT 2025 checklist item description	Reported on page no.
**Title and abstract**			
Title and structured abstract	1a	Identification as a randomised trial	1, 3
	1b	Structured summary of the trial design, methods, results, and conclusions	1
**Open science**			
Trial registration	2	Name of trial registry, identifying number (with URL) and date of registration	n/a
Protocol and statistical analysis plan	3	Where the trial protocol and statistical analysis plan can be accessed	3, 4
Data sharing	4	Where and how the individual de-identified participant data (including data dictionary), statistical code and any other materials can be accessed	11
Funding and conflicts of interest	5a	Sources of funding and other support (e.g., supply of drugs), and role of funders in the design, conduct, analysis and reporting of the trial	11
	5b	Financial and other conflicts of interest of the manuscript authors	11
**Introduction**			
Background and rationale	6	Scientific background and rationale	2
Objectives	7	Specific objectives related to benefits and harms	2
**Methods**			
Patient and public involvement	8	Details of patient or public involvement in the design, conduct and reporting of the trial	3
Trial design	9	Description of trial design including type of trial (e.g., parallel group, crossover), allocation ratio, and framework (e.g., superiority, equivalence, non-inferiority, exploratory)	3
Changes to trial protocol	10	Important changes to the trial after it commenced including any outcomes or analyses that were not prespecified, with reason	6
Trial setting	11	Settings (e.g., community, hospital) and locations (e.g., countries, sites) where the trial was conducted	3

Eligibility criteria	12a	Eligibility criteria for participants	3
	12b	If applicable, eligibility criteria for sites and for individuals delivering the interventions (e.g., surgeons, physiotherapists)	n/a
Intervention and comparator	13	Intervention and comparator with sufficient details to allow replication. If relevant, where additional materials describing the intervention and comparator (e.g., intervention manual) can be accessed	4, 5, 6
Outcomes	14	Pre-specified primary and secondary outcomes, including the specific measurement variable (e.g., systolic blood pressure), analysis metric (e.g., change from baseline, final value, time to event), method of aggregation (e.g., median, proportion), and time point for each outcome	6
Harms	15	How harms were defined and assessed (e.g., systematically, non-systematically)	6
Sample size	16a	How sample size was determined, including all assumptions supporting the sample size calculation	6
	16b	Explanation of any interim analyses and stopping guidelines	n/a
Randomisation:			
Sequence generation	17a	Who generated the random allocation sequence and the method used	5
	17b	Type of randomisation and details of any restriction (e.g., stratification, blocking and block size)	4, 5 and Supplementa ry file
Allocation concealment mechanism	18	Mechanism used to implement the random allocation sequence (e.g., central computer/telephone; sequentially numbered, opaque, sealed containers), describing any steps to conceal the sequence until interventions were assigned	5
Implementation	19	Whether the personnel who enrolled and those who assigned participants to the interventions had access to the random allocation sequence	4
Blinding	20a	Who was blinded after assignment to interventions (e.g., participants, care providers, outcome assessors, data analysts)	5
	20b	If blinded, how blinding was achieved and description of the similarity of interventions	4
Statistical methods	21a	Statistical methods used to compare groups for primary and secondary outcomes, including harms	4, 5, 6

	21b	Definition of who is included in each analysis (e.g., all randomised participants), and in which group	4
	21c	How missing data were handled in the analysis	No missing data
	21d	Methods for any additional analyses (e.g., subgroup and sensitivity analyses), distinguishing prespecified from post-hoc	4
**Results**			
Participant flow, including flow diagram	22a	For each group, the numbers of participants who were randomly assigned, received intended intervention, and were analysed for the primary outcome	3, 4
	22b	For each group, losses and exclusions after randomisation, together with reasons	4, 6
Recruitment	23a	Dates defining the periods of recruitment and follow-up for outcomes of benefits and harms	n/a
	23b	If relevant, why the trial ended or was stopped	n/a
Intervention and comparator delivery	24a	Intervention and comparator as they were actually administered (e.g., where appropriate, who delivered the intervention/comparator, how participants adhered, whether they were delivered as intended [fidelity])	5, 6
	24b	Concomitant care received during the trial for each group	n/a
Baseline data	25	A table showing baseline demographic and clinical characteristics for each group	6
Numbers analysed, outcomes and estimation	26	For each primary and secondary outcome, by group:<br>• the number of participants included in the analysis<br>• the number of participants with available data at the outcome time point<br>• result for each group, and the estimated effect size and its precision (such as 95% confidence interval)<br>• for binary outcomes, presentation of both absolute and relative effect size	4, 5, 6 and supplementary file
Harms	27	All harms or unintended events in each group	6
Ancillary analyses	28	Any other analyses performed, including subgroup and sensitivity analyses, distinguishing pre-specified from post-hoc	7, 8
**Discussion**			

Interpretation	29	Interpretation consistent with results, balancing benefits and harms, and considering other relevant evidence	9-11
Limitations	30	Trial limitations, addressing sources of potential bias, imprecision, generalisability, and, if relevant, multiplicity of analyses	11-12

*© 2025 Hopewell et al. This is an Open Access article distributed under the terms of the Creative Commons Attribution License (https://creativecommons.org/licenses/by/4.0/), which permits unrestricted use, distribution, and reproduction in any medium, provided the original work is properly cited.

- Study design and flowchart details

As presented in the main manuscript's text (under Materials and Methods) we utilized a (3x2x3) within-subjects factorial repeated measures design. The independent variables were Application (Nothing, Weight, Pressure), State (Rest, Fatigue), and Target Angle (30°, 45°, 60°), which respectively constituted Factor 1 (three levels), Factor 2 (two levels), Factor 3 (three levels).

Due to the irreversible nature of acute muscle fatigue within a single testing session, Factor 2 (State) was systematically blocked; all participants completed the Rest conditions first, followed by the fatigue induction protocol, and concluded with the Fatigue conditions second.

To control for order and learning effects, the allocation of Application (Factor 1, nothing, weight, pressure) was randomised as well as the presentation sequence of the three target angles (Factor 3) was randomized for each participant during the Rest state (resulting in one of six possible order permutations, e.g., 45° ▸ 30° ▸ 60°, 30° ▸ 60° ▸ 45°, etc). Each participant was tested across all three target angles in both states.

Crucially, both the Application allocation and the unique, randomized angle sequence established during testing under the Rest state were strictly replicated during the post-fatigue testing block to ensure that any observed changes were due solely to the physiological state change (from rest to fatigue) and not due to an alteration in trial sequence.

As stated in the main manuscript, all participants completed all measurements, i.e. there were no drop-outs, and there were no missing data or harm.

A participant flow diagram was constructed taking into account the CONSORT 2025 Statement [S1] but with methodological adaptations to accurately reflect the sequential, chronological blocks (block 1, Rest vs. Fatigue, Factor 2) and nested within-subject trial randomizations unique to the implemented 3 × 2 × 3 factorial repeated measures design (see **Figure 1**); implementing also the PRISMA 2020 style [S2].

Citations

S1. Hopewell, S.; Boutron, I.; Chan, A.W.; Extier, A.; Gathercole, L.; Giraudeau, B.; Hoffmann, T.C.; Moher, D. CONSORT 2025 statement: updated guideline for reporting parallel group randomised trials. *BMJ* **2025**, *389*, bmj-2024-081124, https://dx.doi.org/10.1136/bmj-2024-081123.

S2. Page MJ, McKenzie JE, Bossuyt PM, Boutron I, Hoffmann TC, Mulrow CD, Shamseer L, Tetzlaff JM, Akl EA, Brennan SE, Chou R, Glanville J, Grimshaw JM, Hróbjartsson A, Lalu MM, Li T, Loder EW, Mayo-Wilson E, McDonald S, McGuinness LA, Stewart LA, Thomas J, Tricco AC, Welch VA, Whiting P, Moher D. The PRISMA 2020 statement: an updated guideline for reporting systematic reviews. BMJ. 2021 Mar 29;372:n71. doi: 10.1136/bmj.n71. PMID: 33782057; PMCID: PMC8005924.
